# Automated detection of electrically evoked stapedius reflexes (eSR) during cochlear implantation

**DOI:** 10.1007/s00405-020-06226-x

**Published:** 2020-08-03

**Authors:** Nora M. Weiss, Attila Óvári, Tobias Oberhoffner, Laurent Demaret, Atabek Bicer, Sebastian Schraven, Karsten Ehrt, Rüdiger Dahl, Armin Schneider, Robert Mlynski

**Affiliations:** 1 Department of Otorhinolaryngology, Head and Neck Surgery, “Otto Körner”, Rostock University Medical Center, Doberaner Strasse 137-139, 18057 Rostock, Germany; 2 Department of Otorhinolaryngology, Head and Neck Surgery, Asklepios Klinik St. Georg, Hamburg, Germany; 3 ARRI Medical GmbH, Türkenstraße 89, 80799 München, Germany

**Keywords:** Electrically evoked stapedius reflex thresholds, eSRT, Cochlea implantation, Objective cochlear implant fitting

## Abstract

**Introduction:**

In cochlear implantation, objective fitting methods are needed to optimize audiological results in small children or patients with poor compliance. Intraoperatively measured electrically evoked stapedius reflexes (eSR) can be used as a marker for the patient’s discomfort level. The aim of this study was to develop and evaluate an automated detection method for eSR and to compare it to the detection rate of the surgeon and independent observers.

**Methods:**

Cochlear implantation using a fully digital surgical microscope was performed. Movements of the stapedius tendon were recorded and analyzed by means of computer vision technique. Differences in eSR elicited by stimulating electrodes at different cochlear locations (basal, middle and apical) were analyzed. The eSR detection rate of the image processing algorithm was compared to the surgeon’s detection rate and to those of two less experienced observers.

**Results:**

A total of 387 electrically impulses were applied. The stimulation of middle turn electrodes showed significantly higher detection rates (50.4%) compared to the basal (40.0%; *p* = 0.001) and apical (43.6%; *p* = 0.03) turn. The software identified significantly more of the applied stimuli (58.4%) compared to the surgeon (46.3%; *p* = 0.0007), the intermediate observer (37.7%; *p* < 0.0001) and the unexperienced observer (41.3%; *p* < 0.0001).

**Conclusion:**

The feasibility of an automated intraoperative software-based detection of eSR is demonstrated. By improving the eSR detection methods and their clinical applicability, their utility in objective cochlear implant fitting may be substantially increased.

## Introduction

In the field of cochlear implantation, efficient objective fitting methods are becoming increasingly important to optimize audiological results in small children or patients with poor compliance. To prevent overstimulation, intraoperatively measured stapedius reflexes can be used as a marker for the patient’s discomfort level [1, 2]. In normal hearing individuals, the stapedius reflex is triggered by sound pressure levels between 70 and 90 dB SPL and leads to a contraction of the stapedius muscle and subsequent movement of the stapedius tendon [3]. During cochlear implantation, the stapedius reflex can be triggered electrically (electrically evoked stapedius reflexes [eSR]) by intracochlear stimulation with the cochlear implant (CI). The movement of the tendon is visualized under the surgical microscope. Stapedius reflexes have been proved to serve as an objective measurement for the patient’s discomfort level during cochlear implantation [4]. Further, stapedius reflexes showed to be a useful research tool investigating the auditory pathway. Reported applications include the determination of the correct cochlear implant electrode position inside the cochlea or the assessment of the cochlear implant integrity [5–7]. Preliminary attempts to measure eSR and to determine the electrically elicited stapedius reflex threshold (eSRT) intraoperatively were performed by means of tympanometry of the contralateral side [8] and using electromyography recordings of the stapedius muscle [9, 10]. However, tympanometry results depend on the morphology and pathology of the middle ear and are not routinely reproducible. Additionally, difficulties may occur in correctly positioning the probe in the external auditory canal. Electromyographic detection of eSR cannot be used routinely in clinical practice by now. The technical limitation is due to background noise and low response amplitudes. Further, this method as introduced by Pau et al. [4] is fairly invasive since a hook electrode needs to be inserted into the stapedius muscle. Therefore, this method may disproportionally extend surgical time and causes additional surgical trauma.

Although intraoperative eSR measurements are considered advantageous compared to postoperative measurements, the eSRT is influenced by the depth of narcosis and may be additionally standardized by intraoperative EEG monitoring [11].

Potential problems during eSR measurements include a poor reproducibility due to the inattention of the observer as well as in patients that miss stapedius reflexes [12]. Furthermore, the visual detection only yields a dichotomous statement (reflex yes/no). The small movements of the ossicles and stapedius tendon after stimulation impede the quantification. Moreover, heartbeat-induced motions, respiratory shift, disturbances of the patient surgical position and vibrations of the microscope can obstruct perception of eSR by the surgeon.

There is a need to optimize the latter issues to reliably use eSR in objective CI fitting. Existing methods with the aim of standardization, increased reproducibility and decreased measurement duration may be improved. A recent study addressing this subject encouraged further development of automatized measurements [13]. Digitization of clinical and surgical methods is discussed as one of the most important innovations in otology [14] and was proved to be a helpful method for teaching purpose and for reproducing intraoperatively recorded surgical steps [15, 16]. Therefore, the aim of this study was to develop and evaluate an automated detection method for eSR and to compare it to the detection rate of the surgeon. The results of this study are intended to support the development of a method for real-time detection of eSR and the determination of eSRT. The digital technique is expected to be less prone to errors such as the surgeon’s attention and the patient movements.

## Materials and methods

### Ethical considerations

The study protocol was approved by the local ethics committee (reference number AZ: 2017-0121) according to the declaration of Helsinki. All participants gave written informed consent.

### Patients selection

A total of ten patients receiving a CI were recruited. All patients were implanted with the same implant and electrode type (Synchrony FLEXsoft, MED-EL GmbH, Innsbruck, Austria). Cochlear implantation was performed under general anesthesia using a fully digital surgical microscope (Arriscope 1.0, ARRI Medical GmbH, Munich, Germany). The electrode was inserted through the round window in every case. All surgeries were performed by a single surgeon (RM).

### Stimulation process

A programming interface (Max Box Programming Interface, MED-EL GmbH, Innsbruck, Austria) with the respective fitting software (Maestro 6.0.1 and 7.0.1, MED-EL GmbH, Innsbruck, Austria) was used for stimulation. Simultaneous to the stimulation, an audio signal was sent to associate the results of reflex detection with the stimuli. The video and audio signals were recorded on a video encoding system (Blackmagic Video Assist 4 K, Blackmagic Design Inc., Fremont, California, USA).

**Fig. 1:**
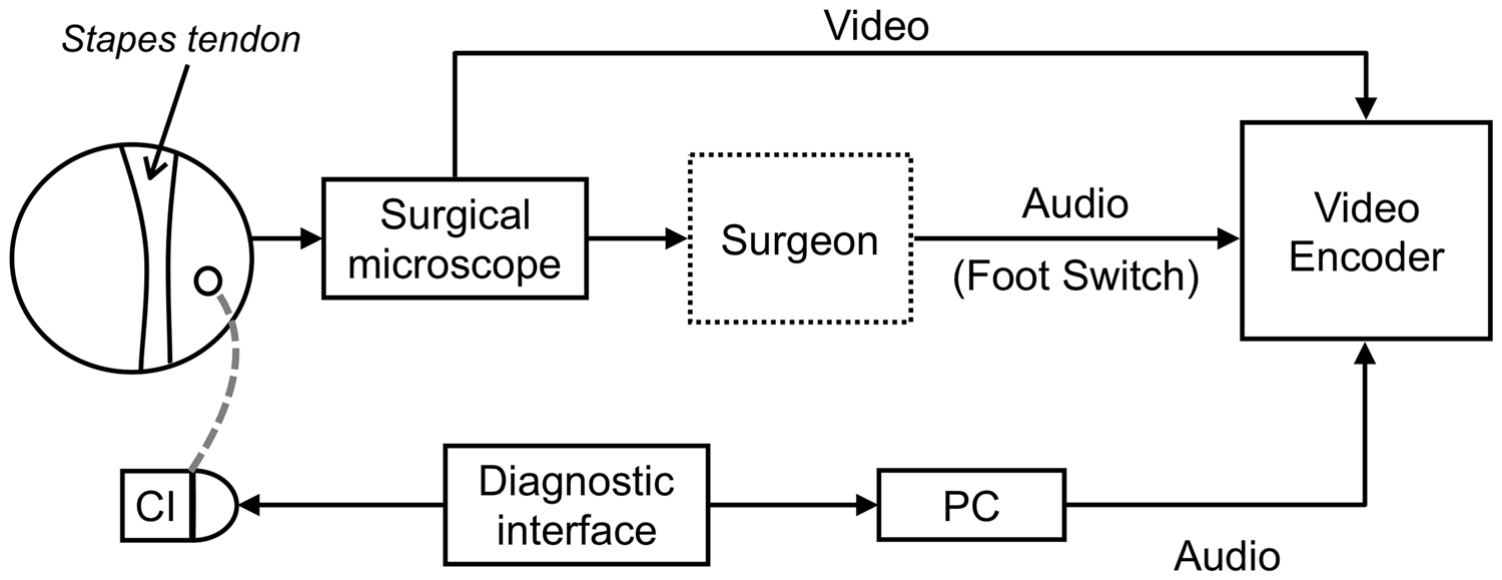
Scheme showing the experimental setup

In all the patients, three electrodes (basal, middle, and apical) were stimulated with increasing stimulation levels until a reflex became visible. After the identification of the eSR, the level was varied to lower and higher values to determine the dynamic range of the eSR. The eSR, detected with an image processing algorithm (see methods subsection “Digital reflex analysis”), were compared with the eSR determined by the surgeon and two independent, less experienced observers (intermediate, unexperienced). Both observers were blinded to the decisions of the surgeon. Figure 1 shows a schematic description of the stimulation, recording and analysis process.

### Surgical microscope

Visualization of the surgical situs was done with the fully digital 3D surgical microscope “Arriscope” (version 1.0, ARRI Medical GmbH, Munich, Germany). In comparison to conventional surgical microscopes, the Arriscope captures the image directly with a complementary metal-oxide semiconductor camera sensor and displays the images on two miniaturized high-density organic light-emitting diode displays mounted in a binocular.

During the eSR recordings, a 210-mm achromatic lens and the maximum zoom level with 6 × magnification was used, resulting in a visual field of 14 × 8 mm^2^. The images were displayed to the surgeon in the digital binoculars of the microscope for visual identification of the eSR.

### Digital reflex analysis

**Fig. 2:**
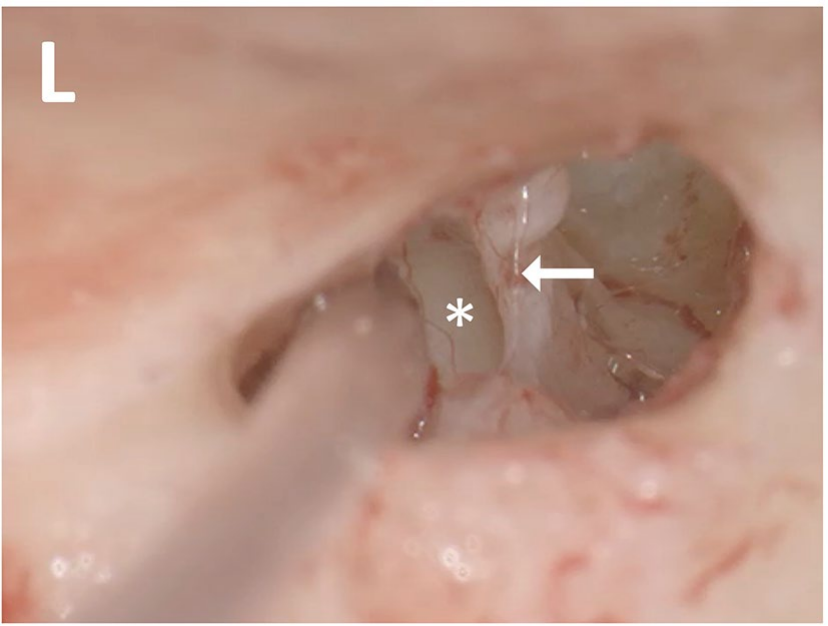
Intraoperative determination of the region of interest (ROI) on the stapes tendon. Arrow: position of the ROI of the stapes movement positioned on the stapes tendon; asterisk: reference ROI positioned at the cochlear promontory

The video data were analyzed using the software Matlab® (version R2016b, The MathWorks, Inc., Natick, MA, USA). To assess the motion at the stapedius tendon, several image processing steps were performed. First, the captured images were stabilized to eliminate influencing movements such as heartbeat or ventilation. In the second step, the stapedius tendon was defined as the region of interest (ROI) and separated from its surroundings (Fig. 2). Within this ROI, several pixels were automatically selected with a geometric distinction to neighborhood pixels. In this given rectangular part of the image, regions were detected by the maximal stable extremal regions (MSER) algorithm [17]. The feature detector MSER analyzed the image by verifying brightness thresholds. Subsequently, regions with similar pixel values were connected. These summarized regions were geometrically defined by an ellipse with a certain width, height and orientation. The center of each ellipse region was defined by coordinate data. The changes in position of these marker points were recalculated for every single frame using the Matlab *PointTracker* function. Each new position of the marker was displayed as a tracker trajectory over the frame number. The motion vectors are computed by the Kanade–Lucas–Tomasi algorithm [18] and then used to calculate the new position of the marker. Small movements were detected along different axes making use of the respective pixel shifts. When exceeding the background noise, the movement of the vector marked at the stapedius tendon was identified being an eSR and was registered by the algorithm. For noise reduction of the tracker trajectory, a Savitzky–Golay filter was implemented [19]. This interpolating signal filter removes unwanted heartbeat artifacts while conserving marker peaks caused by the eSR.

### Observer tests

Postoperatively, the digitally recorded eSRs were analyzed offline by additional observers having less experience in intraoperative detection of eSRs using mobile digital 3D binoculars. These binoculars are similar to those mounted at the digital microscope, thus presenting the same view and quality as it was visible to the surgeon intraoperatively. The raters were blinded concerning the results of each other and the operating surgeon. The results were compared with the detection rate of both the experienced surgeon and the software.

### Statistical analysis

Statistical analyses were performed using Microsoft Excel and Prism (version 8, GraphPad Software, La Jolla, CA, USA). The significance level was set to 0.05. If not otherwise specified, data are presented as mean with standard deviation (SD) or absolute numbers with percentages. For binary variables, a chi-square test was performed. Post hoc analysis was indicated when differences between the groups were identified. In all contingency tables where significant differences were detected between the groups, post hoc testing was performed. After all possible pairwise comparisons were performed between one variable and the others, a Bonferroni correction was conducted to control for a type I error.

## Results

**Fig. 3:**
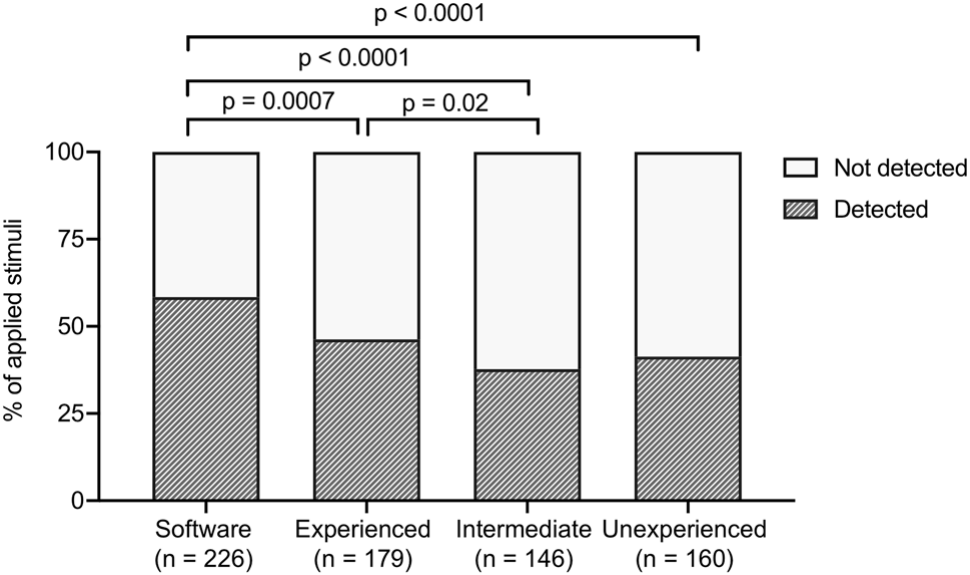
Differences in detection rates among the software and the three observers. N refers to the total number of detected eSR. A total of 387 stimuli were applied

In four patients, no stapedius reflexes were detectable. For this reason, no further testing was performed. In the remaining six patients, a total of 387 stimuli were applied. The software identified eSR in 226/387 (58.4%) of the applied stimuli. The surgeon identified eSR in 179/387 (46.3%), the intermediate observer identified 146/387 (37.7%), and the unexperienced identified 161/387 (41.6%) of the applied eSR (Fig. 3; Table 1).

**Table 1:** Number and percentages of the individual detected stimuli

Patient number	Applied stimuli	Detected reflexes			
		Software	Experienced	Intermediate	Unexperienced
1	56	24 (43%)	22 (39%)	24 (43%)	22 (39%)
2	71	56 (79%)	46 (65%)	29 (41%)	26 (37%)
3	52	22 (42%)	20 (38%)	22 (42%)	20 (38%)
4	73	43 (59%)	34 (47%)	27 (37%)	44 (60%)
5	56	28 (50%)	26 (46%)	17 (30%)	22 (39%)
6	79	53 (67%)	31 (39%)	27 (34%)	27 (34%)
Overall	387	226 (58%)	179 (46%)	146 (38%)	161 (42%)

**Fig. 4:**
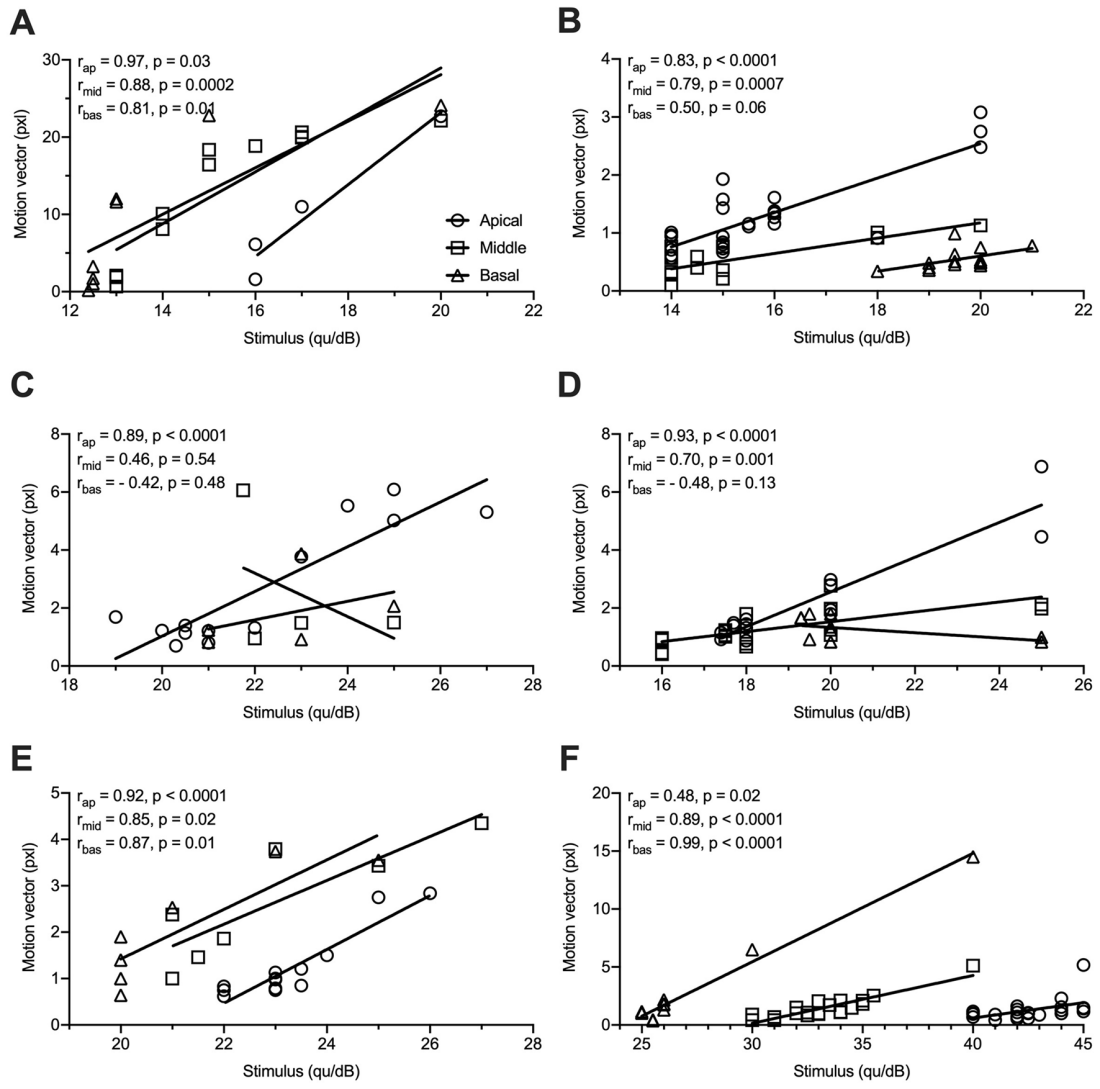
Scatterplot showing the relationship between the stapedius tendon ROI motion vector and the stimulus intensity measured for the different electrodes. **a** Patient 1; **b** Patient 2; **c** Patient 3; **d** Patient 4; **e** Patient 5; **f** Patient 6

There was a statistically significant difference between the detection rate of the software and the experienced surgeon (*p* = 0.0007), between the software and the intermediate observer (*p* < 0.0001), between the software and the unexperienced observer (*p* < 0.0001), and between the experienced surgeon and the intermediate observer (*p* = 0.02). No statistically significant differences between the intermediate and the unexperienced observer (*p* = 0.30) as well as the experienced surgeon and the unexperienced observer (*p* = 0.17) were found. No significant correlation between the stimulus intensity and the length of the motion vector measured in pixels on the stapedius tendon for the overall measurements was found (*p* = 0.1). Regarding the individual patients, correlations between the stimulus intensity and the length of the motion vector measured in pixels on the stapedius tendon were found for the basal, the middle and the apical electrodes of patient 1 (*r*_ap_ = 0.97, *p* = 0.03; *r*_mid_ = 0.88, *p* = 0.0002; *r*_bas_ = 0.81, *p* = 0.01). For patient 2, significant correlations for the middle and apical electrodes but not for the basal electrodes were found (*r*_ap_ = 0.83, *p* < 0.0001; *r*_mid_ = 0.79, *p* = 0.0007; *r*_bas_ = 0.50, *p* = 0.06). For patient 3, only the apical electrode showed significant correlations between the stimulus intensity and the motion vector (*r*_ap_ = 0.89, *p* < 0.0001; *r*_mid_ = 0.46, *p* = 0.54; *r*_bas_ = 0.42, *p* = 0.48). For patient 4, significant correlations were found for the middle and apical electrodes (*r*_ap_ = 0.93, *p* < 0.0001; *r*_mid_ = 0.70, *p* = 0.001; *r*_bas_ = 0.48, *p* = 0.13). Significant correlations for all electrodes were found for patient 5 (*r*_ap_ = 0.92, *p* < 0.0001; *r*_mid_ = 0.85, *p* = 0.02; *r*_bas_ = 0.87, *p* = 0.01) and for patient 6 (*r*_ap_ = 0.48, *p* = 0.02; *r*_mid_ = 0.89, *p* < 0.0001; *r*_bas_ = 0.99, *p* < 0.0001) (Fig. 4).

**Fig. 5:**
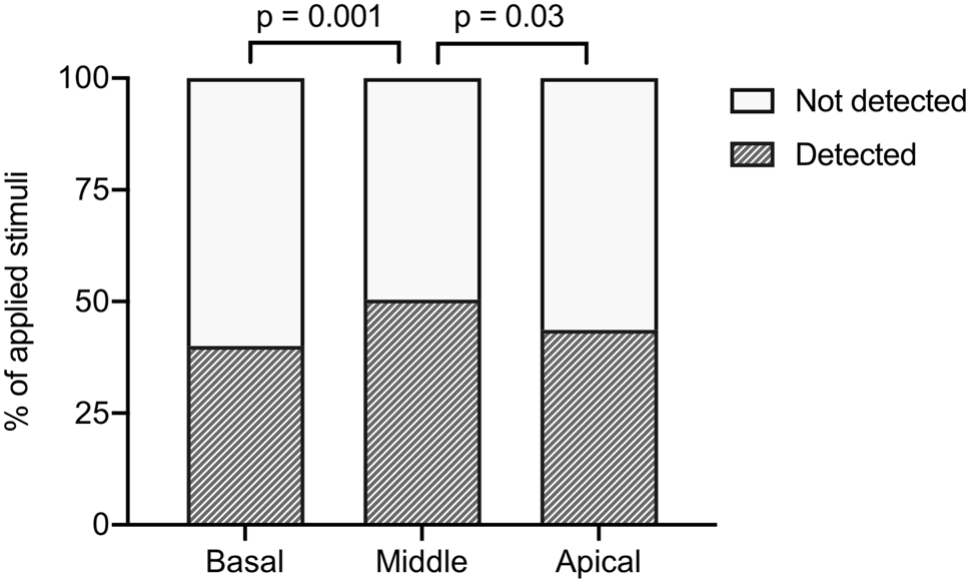
Comparison of the eSR detection rates among the different electrode positions. A total of 387 stimuli were applied

Stimulation of the middle turn electrodes showed significantly higher detection rates (50.4%) compared to the stimulation of the apical (43.6%; *p* = 0.03) and the basal (40.0%; *p* = 0.001) electrodes (Fig. 5). No significant difference between the basal and the apical electrodes was observed (*p* = 0.24).

## Discussion

This is the first study using a fully digital surgical microscope to address eSR measurements. This study confirms the findings of a preliminary study showing that an automatized method to analyze eSR has a higher detection rate than the surgeon [13]. An objective registration of eSR using professional computer vision technology with a tracking software to record the movements of the stapedius tendon was subject of two similar studies [13, 20]. Müller et al. used an endoscope to visualize the stapedius movements [20]. Since the endoscope was held manually, an additional high disturbance rate due to movement artifacts may occur and can be considered disadvantageous. Further standardization to improve the detection rate was encouraged [20]. In the study performed by Weiss et al., the identification points needed to be set manually, and the automatized evaluation was performed postoperatively [13]. This method may complicate the applicability of the method in a routine surgical setting since the surgical time is increased, and the analysis has to be performed after surgery.

To overcome these disadvantages of existing methods, an algorithm that runs on the microscope´s embedded hardware is desirable. Digital microscopes offer new opportunities for software development and clinical investigation. The MSER algorithm used in this study automatically detects brightness-dependent regions in the ROI at the stapedius tendon. Future work based on the findings of this study aims to implement this mechanism into a live mode embedded into the microscope. A live mode enables automated intraoperative eSR measurements and may be used in clinical routine. Further, the algorithm could be expanded to the 3D mode of the surgical microscope. The expanded algorithm would allow for movement detection along three axes, thus enabling an improvement of the detection rate. Moreover, the expanded algorithm could quantify movements of the stapedius tendon.

The results of this study showed no clear correlation between the motion vector and the stimulus intensity which is attributed to the differences in the individual anatomy, the saturation of the reflex and the two-dimensional recording of the vector. Looking at the data of individual patients, significant correlations between the motion vector and the stimulus intensity were found. These findings support the assumption that a quantification of movements is achievable but may be influenced by the individual anatomical structures, the individual eSRT and the determination of the ROI. Thus, this study is the first to address a possible quantification of the reflex. Expanding the technique under the aim of making use of the 3D mode may help to further address this issue. As a side note, since the results of this study showed a clear trend to higher detection rates of the middle turn electrodes, stimulating the middle cochlear turn for eSRT measurements is recommended. This is in line with the finding that the stapedius motoneuron tuning curve exhibits the lowest threshold around 1 kHz [21].

This study has the limitation that only a small number of patients expressing eSR were analyzed. Nevertheless, this number is similar to related studies, and a total of 387 electrical stimuli were considered sufficient for statistical testing and a descriptive analysis of the detection rate.

## Conclusion

This study shows that an automated intraoperative and real-time software-based detection of eSR is possible. The digitization leads to objective and reliable results. The tested algorithm is easily applicable in a routine surgical setting and allows the standardization of the eSR measurement independent of the individual experience of the surgeon. A software that can be implemented into the surgical microscope and can be used for real-time measurements during surgery is currently under development using the acquired results. The present data improve the eSR detection methods. The results support the scientific approaches in objective CI fitting and their clinical applicability.
